# Polarization-Interference Jones Matrix Sensors of Layer-by-Layer Scanning of Polycrystalline Dehydrated Blood Films. Fundamental and Applied Aspects

**DOI:** 10.3390/s25206262

**Published:** 2025-10-10

**Authors:** Oleksandr Ushenko, Yuriy Ushenko, Olexander Bilookyi, Alexander Dubolazov, Mykhaylo Gorsky, Iryna Soltys, Yuriy Rohovy, Viacheslav Bilookyi, Natalia Pavlyukovich, Ivan Mikirin, Oleksandr Salega, Lin Bin, Jun Zheng

**Affiliations:** 1 Institute of Zhejiang University-Taizhou, Taizhou 318000, China; o.ushenko@chnu.edu.ua (O.U.); dbzj@netease.com (J.Z.); 2 Optics and Publishing Department, Chernivtsi National University, 58012 Chernivtsi, Ukraine; a.dubolazov@chnu.edu.ua (A.D.); i.soltys@chnu.edu.ua (I.S.); mikirin.ivan@chnu.edu.ua (I.M.); saleha.oleksandr@chnu.edu.ua (O.S.); dbzj@netease.com (J.Z.); 3 Department of Physics, Shaoxing University, Shaoxing 312399, China; 4 Computer Science Department, Chernivtsi National University, 58012 Chernivtsi, Ukraine; m.gorskiy@chnu.edu.ua; 5 Department of Surgery No. 1, Bucovinian State Medical University, 58012 Chernivtsi, Ukraine; bilookyis@gmail.com (O.B.); slava.bilookyi@bsmu.edu.ua (V.B.); 6 Department of Pathological Physiology, Bucovinian State Medical University, 58012 Chernivtsi, Ukraine; pathophysiology@bsmu.edu.ua; 7 Departament of Internal Medicine, Clinical Pharmacology and Occupational Diseases, Bucovinian State Medical University, 58000 Chernivtsi, Ukraine; natasha.pavlyukovich@bsmu.edu.ua; 8 College of Optical Science and Engineering, Zhejiang University, Hangzhou 310018, China; wjlin@zju.edu.cn

**Keywords:** polarization, interference, holography, Jones matrix image, theziograms of optical anisotropy, blood facies, statistical moments, thyroid, cancer

## Abstract

To date, visual analysis is mainly used to evaluate images of dehydrated films (facies) of biological fluids—microscopy at various magnifications, illumination with white or polarized light, as well as using a dark field. At the same time, important information on the architectonics of optically anisotropic supramolecular networks of facies is unknown (inaccessible). In our work, a model of optical anisotropy of the architectonics of supramolecular networks of blood facies is proposed. Algorithms and a methodology for a new multifunctional method of polarization-interference visualization of the Jones matrix and digital layer-by-layer phase reconstruction of optical anisotropy maps (theziograms) have been developed. As a result, statistically significant markers of oncological changes in the polycrystalline architectonics of supramolecular networks of blood facies samples from healthy donors and patients with papillary thyroid cancer at different stages of the oncological process have been determined and physically analyzed. A comparative study of the diagnostic efficiency of Jones matrix theziography (JT) and Mueller matrix diffusion tomography (MDT) of blood facies samples was conducted within the framework of evidence-based medicine. The main advantages of the Jones matrix method are shown: its multifunctionality (complex detection of birefringence and dichroism), high accuracy of early (stage 1: JM—90.4% and MDT—78.8%) and current (stage 2: JM—96.2% and MDT—88.5%) cancer diagnostics and an excellent level (JM—94.2% and MDT—84.6%) of differentiation of papillary thyroid cancer stages.

## 1. Introduction

One of the most important systems for ensuring the vital activity of the body is a set of biological fluids involved in metabolic processes. In biological liquid (BL), there are high-speed changes in the molecular composition and the nature of the interaction of various components in physiological, extreme, and pathological conditions [1,2,3].

A BL drop lying on a horizontal plane is a convenient model of a self-organizing system for the study of physical–chemical processes whose properties are determined by the composition of dissolved substances in a liquid, external dehydration conditions, and the substrate material. As a result of dehydration of the BL drop, a dry film is formed—a “facia”—the structure of which carries information about the composition and relationships of substances dissolved in BL. By studying such a system at the macroscopic level of its self-organization, the researcher receives information about the behavior of the system at the molecular level [4,5]. To date, visual analysis—microscopy at various magnifications, illumination with white or polarized light, as well as using a dark field—has been mainly used to evaluate images of facies of biological fluids [6,7,8].

The existing microscopic methods for processing the geometric structure of BL images contain only general algorithms, the result of which does not depend on the specifics of the processed images. At the same time, no less important information about the architectonics of supramolecular self-organized networks of BL molecular crystals, and their optical anisotropic properties, which can be extracted by polarization introscopy, turns out to be unknown (inaccessible). The first steps toward polarimetry of the crystalline structure of biological fluid facies were made at the end of the last century [9,10]. This direction was subsequently developed using laser polarimetry techniques; the methods of 2D polarization mapping of microscopic images of facies of blood plasma, blood, urine, and other BL have been developed and experimentally tested using various algorithms (statistical, correlation, wavelet, and Fourier analysis) of data processing [11,12,13,14,15,16,17,18,19,20]. As a result, the first objective criteria (markers) were found, which provided the possibility of differential diagnosis of various pathologies in urology, gynecology, and oncology. However, the sensitivity and progress of these methods were limited by the lack of algorithms for obtaining theziograms of polycrystalline networks of volumetrically inhomogeneous facies of biological fluids.

A new stage in this area of biomedical diagnostics was the successful integration of polarization and interference techniques for mapping laser speckle fields—optical coherence tomography (OCT) [21,22,23,24,25,26,27,28,29]. This technique makes it possible to obtain layer-by-layer coordinate distributions of Jones and Mueller matrix elements, as well as anisotropy maps from small depths (up to 2 mm) of biological tissues. As a result, it was possible to accurately diagnose fibrosis and determine tumor areas with low fibrosis [29]. However, the sensitivity of such systems is limited by the distorting effect of a high level of depolarized laser speckle background on the contrast of polarization images of layers of such tissues. In addition, OCT systems are expensive, have insignificant (8 μm–10 μm) resolution, and do not provide differentiation of benign and malignant tumors, which is important at the early stages of cancer diagnostics. At the same time, the OCT technique has demonstrated new possibilities for three-dimensional polarimetric biomedical diagnostics of pathological changes in biological tissues.

Nevertheless, the results obtained using the OCT technique still remain somewhat empirical. At the moment, there is no systematic information on the capabilities of this method for tissue samples with different optical thickness and depolarizing capacity, morphological structure, and various types of pathology (cancer of various organs and its stages).

From a physical point of view, the solution to this problem consists of establishing criteria for an unambiguous determination of the biological significance of OCT data. The basis for such a solution may be the development and experimental testing of a new laser polarization-interference technology that will expand the functionality of OCT and increase its sensitivity by minimizing the influence of diffuse depolarized background.

The main advantage of the indicated polarization-interference methods is the possibility of selecting components of speckles scattered with different multiplicity by means of layer-by-layer phase scanning of the algorithmically reconstructed object field of complex amplitudes. As a result, conditions are achieved for selecting a single scattered amplitude-phase component, which is unambiguously related to the parameters of the optically anisotropic architectonics of the biological layer.

At present, a small number of publications are known that are aimed at the implementation and diagnostic use of the polarization-interference layer-by-layer visualization techniques of biological preparations of polycrystalline architectonics [30,31,32,33,34,35]. This makes it possible to obtain information on the layer-by-layer statistical structure of tissue biological samples optically anisotropic architectonics, which is inaccessible to traditional 2D matrix polarimetry [9,10,11,12,13,14,15,16,17,18] and OCT techniques [21,22,23,24,25,26,27,28,29]. As a result, the diagnostics and differentiation sensitivity and accuracy of various pathological and necrotic changes in such objects increase. In particular, the high accuracy of benign and malignant tumor differentiation [30,31,32], as well as the possibility of determining the necrotic and traumatic changes in age in the internal organ’s tissues optically anisotropic structure have been demonstrated [33,34,35].

Another important vector of polarization-interference methods development is the study of the biological fluids dehydrated films (facies) polycrystalline architectonics volumetric structure. Such objects are more easily accessible and do not require traumatic biopsy surgery.

In the “pioneering” polarization-interference studies [36] of blood films’ optical anisotropy layered maps the possibility of diagnosing late middle (3 + 3) and low-differentiation prostate cancer (4 + 4; 5 + 3 on the Gleason scale) with high accuracy (90%) was demonstrated. However, the sensitivity of this method was insufficient for diagnosing and differentiating earlier stages of cancer. This circumstance is associated with the following analytical and physical factors:

- The Mueller matrix technique has been developed and is analytically adequate for measuring the coordinate distributions of the magnitude of polarization-filtered intensity arrays of scattered object fields of biological preparations [36,37,38,39,40,41,42,43,44].
- The specified vector parametric approach does not provide the possibility of implementing a direct Fourier reconstruction of layer-by-layer distributions of complex amplitudes of the scattered laser field.

Based on the above, it can be concluded that the 3D technique of Mueller matrix tomography of the polycrystalline structure of biological preparations using laser probing requires further development, both in terms of sensitivity, as well as in analytical and instrumental aspects.

Our work uses a different methodology that is more adapted to polarization-inhomogeneous laser fields. Its main differences are:

- Adequate analytical Jones matrix formalism for the direct description of the processes of formation of polarization structure of amplitude-phase coherent object fields of polycrystalline blood films.
- Application of digital Fourier reconstruction and layer-by-layer phase scanning of Jones matrix images of complex amplitudes with subsequent reconstruction of distributions of linear and circular birefringence-dichroism of blood film samples.

Based on the above, it can be assumed that the proposed polarization-interference Jones matrix sensor of changes in optical anisotropy of blood film samples will provide greater accuracy and sensitivity in early cancer diagnostics. Therefore, we took advantage of these advantages in order to determine the criteria for differential diagnostics of earlier stages of cancer using stage 1 papillary thyroid cancer as an example.

The relevance of this task is due to the fact that traditional digital microscopy techniques [45] aimed at studying thyroid nodule biopsy with subsequent obtaining of intensity distribution histograms have become an addition to histological methods. On this basis, the possibility of differential diagnosis of thyroid nodules as benign (follicular adenoma) or malignant (papillary carcinoma) was demonstrated.

New objective quantitative technologies with high spatial resolution have become a qualitative development of digital histology [46]. First of all, these include polarization-sensitive second harmonic generation (PSHG) microscopy methods [47,48]. These techniques have demonstrated the ability to differentiate cancerous or diseased tissues by analyzing circular dichroism and modulating laser polarization states [49].

However, at this point in time, there is no systematic quantitative information on the effect of light scattering on the accuracy of differential diagnostics within representative samples of histological biopsy specimens of thyroid tissue.

The question of the possibility of using the PSHG technique in studying the relationships between the optically anisotropic architecture of blood films and pathological conditions of the thyroid gland remains open.

The goal of our work is development of algorithms and experimental testing of a new multifunctional method of polarization-interference visualization of the Jones matrix and digital layer-by-layer phase reconstruction of optical anisotropy maps (theziograms) of supramolecular networks of dehydrated blood films (phases) by determining statistically significant markers characterizing the reconstructed layer-by-layer theziograms of birefringence and dichroism for early diagnosis (stage 1) and differentiation of stages (stage 1 and stage 2) of papillary thyroid cancer.

## 2. Basic Equations and Theoretical Remarks

This part of the article is devoted to the theoretical substantiation of the multifunctional method of Jones matrix theziography of optically anisotropic polycrystalline architectonics of dehydrated blood films. Analytical interrelations between linear and circular birefringence and dichroism of supramolecular networks and matrix elements, the value of which is determined by the orthogonal components of complex amplitudes of the object field of blood facies, are determined. This ensured the development of principles for layer-by-layer reconstruction of the parameters of optical anisotropy of the polycrystalline structure of biological films.

According to traditional concepts [50,51,52,53], single scattering approximations by a polycrystalline film of any biological fluid can be represented as a sequence of partial layers with six different optical anisotropy types: linear and circular birefringence ($LB_{\left(0.90\right);\left(45,135\right)},CB_{⊗,⊕}$) and dichroism ($LD_{\left(0.90\right);\left(45,135\right)},CD_{⊗,⊕}$). These $LD_{0.90}$, $LD_{45,135}$, $LB_{0.90}$, and $LB_{45,135}$—“linear dichroism—birefringence”; $CD_{⊗;⊕}$ and $CB_{⊗;⊕}$—“circular dichroism—birefringence” of the biological layer of the optically anisotropic component for linearly ($0^{0}÷90^{0}$ and $45^{0}÷135^{0}$) and circularly right—(⊗) and left—(⊕) polarized orthes.

The mechanisms of linear birefringence and dichroism (structural anisotropy $LB_{\left(0.90\right);\left(45,135\right)},LD_{\left(0.90\right);\left(45,135\right)}$) are inherent in the dendrite polycrystalline networks, which are formed by needle-like crystals [9,10,11].

Molecular complexes of spherulite crystals have optical activity ($CB_{⊗,⊕}$) and circular dichroism ($CD_{⊗,⊕}$) [12,13,14,15,16].

An analytical form of the generalized Jones matrix of a polycrystalline medium with complex anisotropy was found in [40,41]

(1) $$\begin{array}{c} \left\{W\right\}=\left\|\begin{array}{cc} w_{11} & w_{12}\\ w_{21} & w_{22} \end{array}\right\|=\\ =\left\|\begin{array}{cc} \cos0.5\left|Q\right|-i\frac{B_{0;90}}{\left|Q\right|}\sin0.5\left|Q\right| & \frac{\left(A_{⊗;⊕}-iB_{45;135}\right)}{\left|Q\right|}\sin0.5\left|Q\right|\\ -\frac{\left(A_{⊗;⊕}+iB_{45;135}\right)}{\left|Q\right|}\sin0.5\left|Q\right| & \cos0.5\left|Q\right|+i\frac{LB_{0;90}}{\left|Q\right|}\sin0.5\left|Q\right| \end{array}\right\| \end{array}$$

here Q—generalized anisotropy vector

(2) $$Q=Q\left(B_{0;90},B_{45;135};-A_{⊗;⊕}\right)$$

where

(3) $$\left\{\begin{array}{c} B_{0;90}=LB_{0;90}-iLD_{0;90};\\ B_{45;135}=LB_{45;135}-iLD_{45;135};\\ A_{⊗;⊕}=CB_{⊗;⊕}-iCD_{⊗;⊕}. \end{array}\right.$$

from (2), (3) we obtain an explicit form of the vector modulus V

(4) $$\begin{array}{cc} \left|Q\right|=(\left|B_{0;90}\right|+ & \left|B_{45;135}\right|+\left|A_{⊗;⊕}\right|{)}^{0.5}\\ & =(\left(L{D^{2}}_{0;90}+L{B^{2}}_{0;90}\right)+\left(L{D^{2}}_{45;135}+L{B^{2}}_{45;135}\right)\\ & +\left(C{D^{2}}_{⊗;⊕}+C{B^{2}}_{⊗;⊕}\right){)}^{0.5}\\ & ={\left(\left({LB}^{2}+{CB}^{2}\right)+\left({LD}^{2}+{CD}^{2}\right)\right)}^{0.5} \end{array}$$

here

(5) $$LB={\left(LB_{0;90}^{2}+LB_{45;135}^{2}\right)}^{0.5}$$

(6) $$LD={\left(LD_{0;90}^{2}+LD_{45;135}^{2}\right)}^{0.5}$$

The algorithm for calculating the elements of the generalized Jones matrix $\left\{W\right\}$ (Equation (1)) includes the irradiation of a polycrystalline **BF** sample by linearly polarized beams with azimuths $0^{0}$ (Jones vector $\left(\begin{array}{c} 1\\ 0 \end{array}\right)$) and $90^{0}$ (Jones vector $\left(\begin{array}{c} 0\\ 1 \end{array}\right)$)

(7) $$E^{0}=\left(\begin{array}{c} E_{x}^{0}\\ E_{y}^{o} \end{array}\right)=\left\|\begin{array}{cc} w_{11} & w_{12}\\ w_{21} & w_{22} \end{array}\right\|\left(\begin{array}{c} 1\\ 0 \end{array}\right)\Rightarrow \left(\begin{array}{c} w_{11}\\ w_{21} \end{array}\right)\Rightarrow \left\{\begin{array}{c} E_{x}^{0}=\left\|\begin{array}{cc} 1 & 0\\ 0 & 0 \end{array}\right\|\left(\begin{array}{c} w_{11}\\ w_{21} \end{array}\right)\Rightarrow w_{11};\\ E_{y}^{0}=\left\|\begin{array}{cc} 0 & 0\\ 0 & 1 \end{array}\right\|\left(\begin{array}{c} w_{11}\\ w_{21} \end{array}\right)\Rightarrow w_{21}; \end{array}\right.$$

(8) $$E^{90}=\left(\begin{array}{c} E_{x}^{90}\\ E_{y}^{90} \end{array}\right)=\left\|\begin{array}{cc} w_{11} & w_{12}\\ w_{21} & w_{22} \end{array}\right\|\left(\begin{array}{c} 0\\ 1 \end{array}\right)\Rightarrow \left(\begin{array}{c} w_{12}\\ w_{22} \end{array}\right)\Rightarrow \left\{\begin{array}{c} E_{x}^{90}=\left\|\begin{array}{cc} 1 & 0\\ 0 & 0 \end{array}\right\|\left(\begin{array}{c} w_{12}\\ w_{22} \end{array}\right)\Rightarrow w_{12};\\ E_{y}^{90}=\left\|\begin{array}{cc} 0 & 0\\ 0 & 1 \end{array}\right\|\left(\begin{array}{c} w_{12}\\ w_{22} \end{array}\right)\Rightarrow w_{22}. \end{array}\right.$$

Based on (1)–(8), theoretical relationships between the parameters of linear and circular birefringence-dichroism and optically anisotropic **BF** layer Jones matrix $\left\{J\right\}$ elements are established

(9) Q=2arccos⁡0.5Re⁡w11+Re⁡w22

**Birefringence**

(10) LB=Q2sin⁡0.5QIm⁡w22−Im⁡w112+Im⁡w12+Im⁡w2120.5;CB⊗;⊕=Q2sin⁡0.5QIm⁡w12−Im⁡w21;

**Dichroism**

(11) LD=Q2sin⁡0.5QRe⁡w22−Re⁡w112+Re⁡w12+Re⁡w2120.5;CD⊗;⊕=Q2sin⁡0.5QRe⁡w12−Re⁡w21.

As a result, we obtain the following expressions for the BF polycrystalline structure Jones matrix theziography

(12) $$\left|Q\right|=2\arccos\left[0.5\left(\left|E_{x}^{0}\right|\cos\left(\varphi_{x}^{0}\right)+\left|E_{y}^{90}\right|\cos\left(\varphi_{y}^{90}\right)\right)\right]$$

**Birefringence**

(13) $$\left\{\begin{array}{c} LB=\frac{\left|Q\right|}{2\sin0.5\left|Q\right|}{\left({\left(\left|E_{y}^{90}\right|\sin\varphi_{y}^{90}-\left|E_{x}^{0}\right|\sin\varphi_{x}^{0}\right)}^{2}-{\left(\left|E_{y}^{0}\right|\sin\varphi_{y}^{0}-\left|E_{x}^{90}\right|\sin\varphi_{x}^{90}\right)}^{2}\right)}^{0.5};\\ CB_{⊗;⊕}=\frac{\left|Q\right|}{2\sin0.5\left|Q\right|}\left(\left|E_{x}^{90}\right|\sin\varphi_{x}^{90}-\left|E_{y}^{0}\right|\sin\varphi_{y}^{0}\right). \end{array}\right.$$

**Dichroism**

(14) $$\left\{\begin{array}{c} LD=\frac{\left|Q\right|}{2\sin0.5\left|Q\right|}{\left({\left(\left|E_{y}^{90}\right|\cos\varphi_{y}^{90}-\left|E_{x}^{0}\right|\cos\varphi_{x}^{0}\right)}^{2}-{\left(\left|E_{y}^{0}\right|\cos\varphi_{y}^{0}-\left|E_{x}^{90}\right|\cos\varphi_{x}^{90}\right)}^{2}\right)}^{0.5};\\ CD_{⊗;⊕}=\frac{\left|Q\right|}{2\sin0.5\left|Q\right|}\left(\left|E_{x}^{90}\right|\cos\varphi_{x}^{90}-\left|E_{y}^{0}\right|\cos\varphi_{y}^{0}\right) \end{array}\right.$$

In what follows, expressions (13)–(14) will be called Jones matrix theziogram

(15) Ta,b=LBIm⁡wikCB⊗;⊕Im⁡wik;LDRe⁡wik;CD⊗;⊕Re⁡wik

Note that the theziograms (15) of the **BF** are obtained in the single scattering approximation and, therefore, they unambiguously display the optical properties of their polycrystalline structure. However, for most real **BF**, such an approximation is not performed or is difficult to achieve. As a result, the set of theziograms (15) displays integrally averaged information about the optical anisotropy maps of the facies under study.

In our work, we will consider the possibility of overcoming this problem. It is based on the principles of Mueller polarization-interference reconstruction and phase scanning biological layers object complex amplitude field technique [28,29,30,31,32,33,34,35].

As we have already noted, the peculiarity of our method in the implementation of the technique of phase scanning of laser object fields of polycrystalline blood films is the application of the Jones matrix formalism (relations (1)–(9)) for the reconstruction of optical anisotropy theziograms (relations (10)–(15)).

The main experimental toolkit of this approach is the sequential implementation of:

- complex interference registration and polarization filtering of the object field of blood facies;
- digital Fourier reconstruction of the amplitude-phase structure of the object field;
- step-by-step phase scanning of distributions of complex amplitudes;
- algorithmic reconstruction of Jones matrix theziograms of “birefringence-dichroism” in a set of phase planes.

The methodological implementation of this polarization-interference technique is illustrated in the following section of the article.

## 3. Three-Dimensional Jones Matrix Scanning Method

1. Two states of linear polarization are sequentially formed in the “irradiating” (Ir) and “reference” (Re) parallel laser beams—Ir(00)−Re⁡(00)≡p and Ir(900)−Re⁡(900)≡r.
2. For each of the polarization states (p and r), two partial interference patterns are recorded through the polarizer–analyzer with the transmission plane orientation at angles $\begin{array}{cc} \Omega =0^{0}; & \Omega =90^{0} \end{array}$.
3. For each partial interference distribution, we perform a two-dimensional discrete Fourier transform $\Phi \left(\upsilon ,\nu \right)$ [10,12]:
  (16) $$\left\{\begin{array}{c} {w_{11}\equiv \Phi }_{x}^{0}=\frac{1}{A\times B}\sum_{a=0}^{A-1}\sum_{b=0}^{B-1}E_{x}^{0}{\left(E_{x}^{0}\right)}^{*}\left(a,b\right)\exp\left[-i2\pi \left(\frac{a\times \upsilon }{A}+\frac{b\times \nu }{B}\right)\right];\\ {w_{21}\equiv \Phi }_{y}^{0}=\frac{1}{A\times B}\sum_{a=0}^{A-1}\sum_{b=0}^{B-1}E_{y}^{0}{\left(E_{y}^{0}\right)}^{*}\left(a,b\right)\exp\left[-i2\pi \left(\frac{a\times \upsilon }{A}+\frac{b\times \nu }{B}\right)\right];\\ {w_{12}\equiv \Phi }_{x}^{90}=\frac{1}{A\times B}\sum_{a=0}^{A-1}\sum_{b=0}^{B-1}E_{x}^{90}{\left(E_{x}^{90}\right)}^{*}\left(a,b\right)\exp\left[-i2\pi \left(\frac{a\times \upsilon }{A}+\frac{b\times \nu }{B}\right)\right];\\ {w_{22}\equiv \Phi }_{y}^{90}=\frac{1}{A\times B}\sum_{a=0}^{A-1}\sum_{b=0}^{B-1}E_{y}^{90}{\left(E_{y}^{90}\right)}^{*}\left(a,b\right)\exp\left[-i2\pi \left(\frac{a\times \upsilon }{A}+\frac{b\times \nu }{B}\right)\right] \end{array}\right.$$
  Digital Fourier transforms (16) for determining the set of Jones matrix theziograms Ta,b=LBRe⁡wikCB⊗;⊕Re⁡wik;LDIm⁡wik;CD⊗;⊕Im⁡wik can be rewritten in the following form
  (17) $$Re\left\{\begin{array}{c} {w_{11}\equiv \Phi }_{x}^{0}=\frac{1}{A\times B}\sum_{a=0}^{A-1}\sum_{b=0}^{B-1}E_{x}^{0}\left(a,b\right){\left(E_{x}^{0}\right)}^{*}\left(a,b\right)\cos\left(\frac{a\times \upsilon }{A}+\frac{b\times \nu }{B}\right);\\ {w_{21}\equiv \Phi }_{y}^{0}=\frac{1}{A\times B}\sum_{a=0}^{A-1}\sum_{b=0}^{B-1}E_{y}^{0}\left(a,b\right){\left(E_{y}^{0}\right)}^{*}\left(a,b\right)\cos\left(\frac{a\times \upsilon }{A}+\frac{b\times \nu }{B}\right);\\ {w_{12}\equiv \Phi }_{x}^{90}=\frac{1}{A\times B}\sum_{a=0}^{A-1}\sum_{b=0}^{B-1}E_{x}^{90}\left(a,b\right){\left(E_{x}^{90}\right)}^{*}\left(a,b\right)\cos\left(\frac{a\times \upsilon }{A}+\frac{b\times \nu }{B}\right);\\ {w_{22}\equiv \Phi }_{y}^{90}=\frac{1}{A\times B}\sum_{a=0}^{A-1}\sum_{b=0}^{B-1}E_{y}^{90}\left(a,b\right){\left(E_{y}^{90}\right)}^{*}\left(a,b\right)\cos\left(\frac{a\times \upsilon }{A}+\frac{b\times \nu }{B}\right) \end{array}\right.$$
  (18) $$Im\left\{\begin{array}{c} {w_{11}\equiv \Phi }_{x}^{0}=\frac{1}{A\times B}\sum_{a=0}^{A-1}\sum_{b=0}^{B-1}E_{x}^{0}{\left(E_{x}^{0}\right)}^{*}\left(a,b\right)\sin\left(\frac{a\times \upsilon }{A}+\frac{b\times \nu }{B}\right);\\ {w_{21}\equiv \Phi }_{y}^{0}=\frac{1}{A\times B}\sum_{a=0}^{A-1}\sum_{b=0}^{B-1}E_{y}^{0}{\left(E_{y}^{0}\right)}^{*}\left(a,b\right)\sin\left(\frac{a\times \upsilon }{A}+\frac{b\times \nu }{B}\right);\\ {w_{12}\equiv \Phi }_{x}^{90}=\frac{1}{A\times B}\sum_{a=0}^{A-1}\sum_{b=0}^{B-1}E_{x}^{90}{\left(E_{x}^{90}\right)}^{*}\left(a,b\right)\sin\left(\frac{a\times \upsilon }{A}+\frac{b\times \nu }{B}\right);\\ {w_{22}\equiv \Phi }_{y}^{90}=\frac{1}{A\times B}\sum_{a=0}^{A-1}\sum_{b=0}^{B-1}E_{y}^{90}{\left(E_{y}^{90}\right)}^{*}\left(a,b\right)\sin\left(\frac{a\times \upsilon }{A}+\frac{b\times \nu }{B}\right) \end{array}\right.$$
  where $E_{x,y}^{0.90}—$ complex amplitude orthogonal components for different orientations $\begin{array}{cc} \Omega =0^{0}; & \Omega =90^{0} \end{array}$ *—denotes the complex conjugation operation; $\left(\upsilon ,\nu \right)$ they are the spatial frequencies and $\left(a=1120,b=960\right)\;$are the quantity of pixels of the CCD camera.
4. The digital Fourier transforms (relations (16)–(18)) results are used to obtain complex amplitude distributions according to the following algorithms:
  (19) $$\left\{\begin{array}{c} E_{0^{0}}\to \left|E_{x}^{0}\left(\Omega =0^{0}\right)\right|;\\ E_{90^{0}}\to \left|E_{x}^{90}\left(\Omega =90^{0}\right)\right|\exp\left(i\left(\varphi_{x}^{90}-\varphi_{x}^{0}\right)\right); \end{array}\right.$$
  (20) $$\left\{\begin{array}{c} E_{0^{0}}\to \left|E_{y}^{0}\left(\Omega =0^{0}\right)\right|;\\ E_{90^{0}}\to \left|E_{y}^{90}\left(\Omega =90^{0}\right)\right|\exp\left(i\left(\varphi_{y}^{90}-\varphi_{y}^{0}\right)\right). \end{array}\right.$$
5. Using stepwise (Δφ) phase ($\varphi_{k}$) reconstructed complex amplitudes (relations (19), (20)) scanning and using algorithms (13), (14) we obtain Jones matrix theziograms Ta,b=LBRe⁡wikCB⊗;⊕Re⁡wik;LDIm⁡wik;CD⊗;⊕Im⁡wik for differently scattered components of the BF object field.
6. The resulting set of **BF** Jones matrix theziograms $T\left(a,b\right)$ was analyzed in a statistical approach using the following algorithms to calculate the mean ($Z_{1}$), variance ($Z_{2}$), skewness ($Z_{3}$), and kurtosis ($Z_{4}$) [9,10,11,12,13,14,15,28,29,30,31,32,33,34,35]
  (21) $$\begin{array}{c} Z_{1}=\frac{1}{K}\sum_{j=1}^{K}T_{j};\\ Z_{2}=\sqrt{\frac{1}{K}\sum_{j=1}^{K}{\left(T-Z_{1}\right)}_{j}^{2};}\\ Z_{3}=\frac{1}{Z_{2}^{3}}\frac{1}{K}\sum_{j=1}^{K}{\left(T-Z_{1}\right)}_{j}^{3};\\ Z_{4}=\frac{1}{Z_{2}^{4}}\frac{1}{K}\sum_{j=1}^{K}{\left(T-Z_{1}\right)}_{j}^{4}, \end{array}$$
  where $K\left(a,b\right)$—CCD pixels quantity.

The presented analytical relations (16)–(21) constituted the algorithmic basis of the methodology of multifunctional Jones matrix theziography of polycrystalline blood films.

The results of its experimental implementation are presented in the next section of the article.

## 4. Experimental Results and Discussion

This study was conducted in accordance with the principles of the Declaration of Helsinki, and in compliance with the International Conference on Harmonization Good Clinical Practice, and local regulatory requirements. Ethical approval was obtained from the Ethics Committee (protocol №7, 16 May 2024) of the Bukovinian State Medical University (Chernivtsi, Ukraine), and written informed consent was obtained from all subjects prior to study initiation.

The fundamental aspect of the research is considered, which is related to the fact that in the process of dehydration and molecular self-assembly, various architectonics and optical anisotropy of polycrystalline networks of various constituent **BF** are formed [54,55,56]. The obtained data on the polarization manifestations of the optical properties of such networks can be used to determine the totality of physical and quantitative relationships between the topology of optical anisotropy maps and the specifics of the biological crystal’s ensembles spatial-angular structure. For this purpose, polarization-interference mapping, analytical reconstruction, statistical processing, and physical analysis of integral and layered Jones matrix theziograms of linear and circular birefringence and dichroism of healthy donors **BF** were carried out.

### 4.1. Objects of Investigations

In our work, we will consider **BF**, which consists of two main parts:

- blood plasma;
- shaped elements.

*Blood plasma*. It is a liquid of a complex structure, a heterogeneous colloidal polymer solution. Traditionally, the molecular components (monomers of albumin (~60%), globulin (~40%), fibrinogen, hemoglobin) of blood plasma are effectively studied by biochemical methods. However, there are currently insufficient effective research methods to study supramolecular optically anisotropic (dimers, trimers, and monomers) protein structures (100 nm–2000nm) of blood plasma [54,55,56].

Three zones of different thickness h are distinguished in the structure of the blood plasma surface:

- edge (polycrystalline albumin layer with linear birefringence and dichroism, h ~50μm–60μm);
- transitional (outer and inner layers of optically isotropic cubic crystals of the Na–Cl salt, intermediate globulin layer with circular birefringence and dichroism, h ~25 μm–30 μm);
- the central (outer layer of cubic crystals of salt Na–Cl, h ~5 μm–15μm).

Such an image of plasma binding possesses mainly a dendritic supramolecular polycrystalline network with structural anisotropy (“LB−LD”) and insignificant optical activity (“CB−CD”). In the future, such a network will be called dendritic.

*The shaped elements* are erythrocytes, platelets, and leukocytes with circular birefringence and dichroism. As a result, a spherulite polycrystalline network is formed (“CB−CD”).

**Figure 1:**
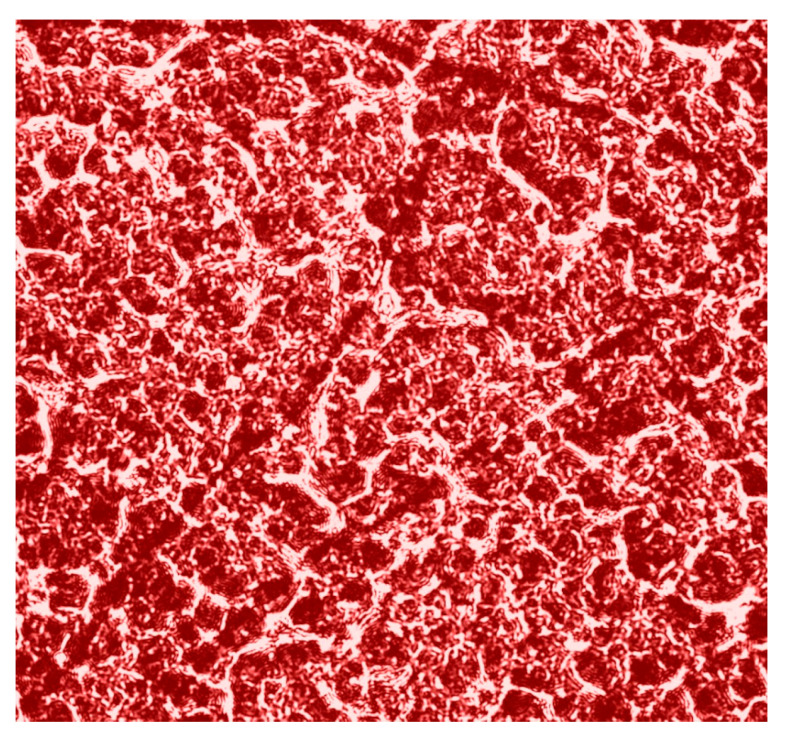
Microscopic image (×4) of the polycrystalline structure of blood facies.

Experimental samples of blood facies were provided by the Department of Surgery № 1 of the Bukovinian State Medical University (as example—Figure 1).

**BF** samples were obtained by applying a liquid drop to a glass substrate at room temperature (t=22 °C) and drying for 24 h. We reduced the volume of a liquid drop to 0.035–0.004 μL. As a result of blood dehydration, blood facies were formed with an average height of up to 60 μm and a diameter of up to 3–4 mm.

The extinction coefficient ($\tau ,cm^{-1}$) of **BF** layers was measured according to the standard photometry attenuation illuminating beam intensity method [57] using an integral light-scattering sphere [58]. The measurement of the **BF** sample’s integral depolarization degree (Λ,%) was carried out in the standard Mueller matrix polarimeter scheme [12,13,14,15,16].

Optical-geometric parameters of samples **BF** are presented in Table 1.

The number of samples to ensure statistical significance (representativeness) of the blood facies group was determined by the cross-validation method [59]. The standard deviation $\sigma^{2}\;$of the statistical moments $Z_{i=1;2;3;4}\left(n\right)$, which characterize the distribution of the Jones matrix theziograms $T\left(\varphi_{k},(a,b)\right)\;$parameter values, was determined. The specified number (n=26) of samples provided the level $\sigma^{2}\leq 0.025$. This standard deviation corresponds to a confidence interval of p≺0.05, which demonstrates the statistical reliability of the 3D Jones matrix mapping method.

In order to study the volumetric optically anisotropic polycrystalline structure of blood facies samples, the polarization-interference method of mapping laser object fields was used. For the purpose of experimental implementation of theoretical algorithms of laser layer-by-layer Jones matrix theziography (relationships (1)–(20)) of supramolecular networks of blood facies samples (Table 1), a Mach-Zehnder interferometer platform was used, a photograph and a brief description of the laboratory implementation of which are presented in the next part of the article.

### 4.2. The Experimental Setup

**Figure 2:**
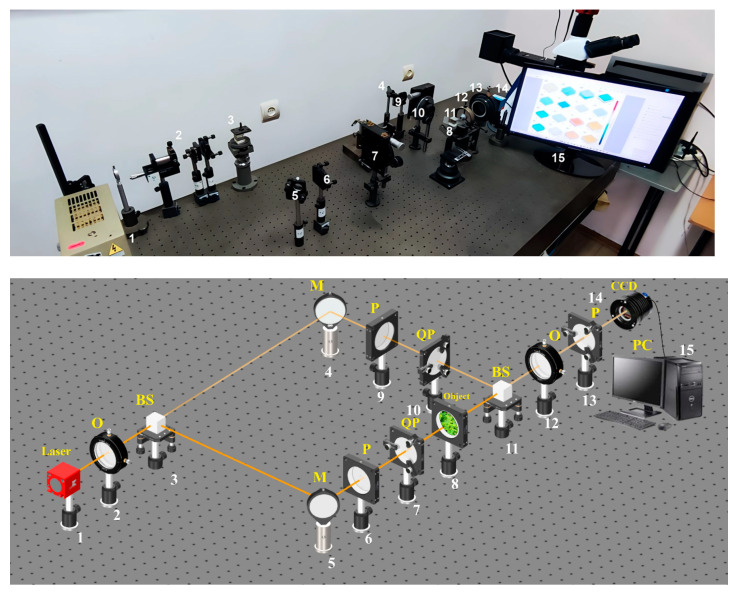
Optical scheme for **BF** polarization-interference mapping: 1—He–Ne laser; 2—collimator—“O”; 3, 11—beam splitters—“BS”; 4, 5—mirrors—“M”; 6, 9, and 13—polarizer’s “P”; 7, 10—quarter wave plates—“QP”; 8—object; 12—polarization objective—“O”; 14—digital camera—“CCD”; 15—personal computer—“PC”.

Photograph and optical scheme of the tabletop experimental setup of the Mach-Zehnder polarization interferometry scheme is shown in Figure 2 [28,29,30,31,32,33,34,35].

The parallel $Ø=2\times 10^{3}\mu m$ beam of He–Ne (λ=0.6328 μm) laser 1 formed by spatial-frequency filter 2, with 50% of beam splitter 3 is divided into “object” and “reference” ones. The “object” beam is guided by rotating mirror 5 through polarizing filters 6–7 toward BF sample 8. The polarization-inhomogeneous image of BF 8 is then projected onto digital camera 14 plane using the strain-free objective 12.

The “reference” beam is directed by mirror 4 through the polarization filter 9–10 into the **BF** 8 polarization-inhomogeneous image plane. As a result, an interference pattern is formed, the coordinate intensity distribution of which is recorded by digital camera 14 through polarizer 13. Before carrying out the measurements of **BF**, the experimental device passed metrological certification with the model objects (“clean air”, “linear polarizer”, “phase plates 0”.25” λ”, “0”.5” λ”) introduction. As 50 measurements result for each object, the optical anisotropy errors were determined $∆T=5\times 10^{-5}rad$.

The next part of the article is devoted to the analysis of group experimental measurements of polarization-filtered interference distributions of image intensity of blood facies samples with the subsequent algorithmic restoration of a set of layered Jones matrix theziograms of optically anisotropic architectonics of supramolecular networks.

### 4.3. Jones Matrix Theziography of Phase Anisotropy of Polycrystalline Blood Films

**Figure 3:**
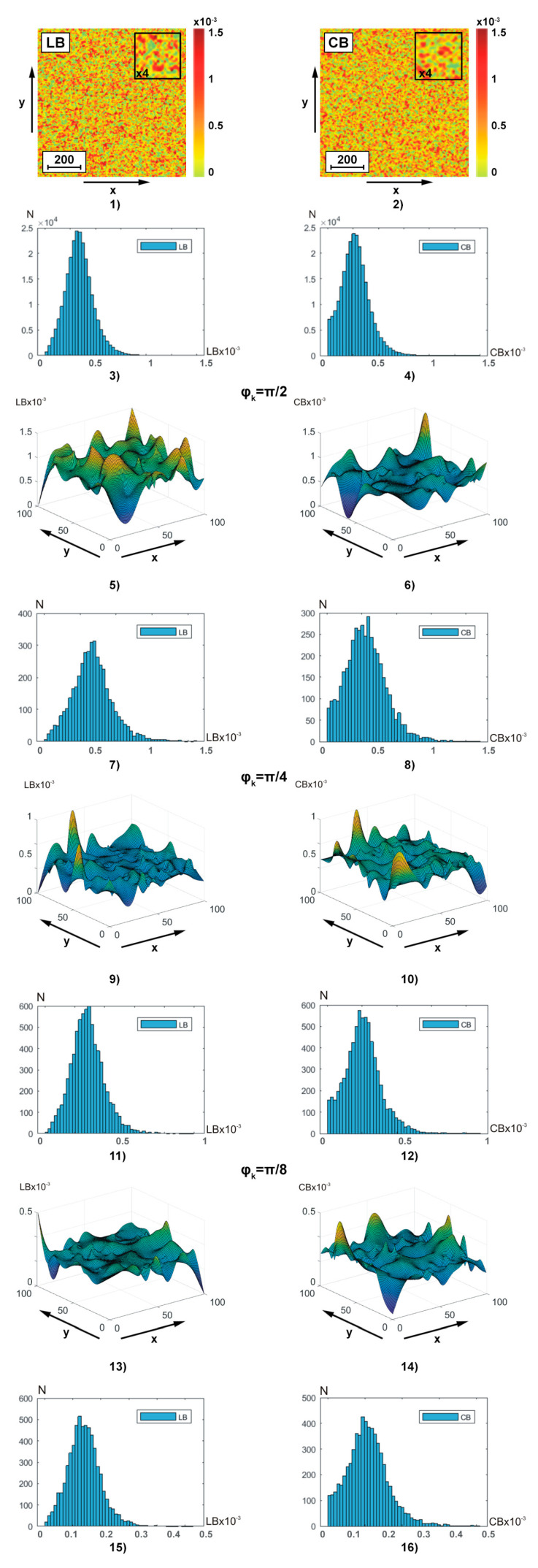
Two-dimensional distributions and histograms of integral ((1)–(4)) and layer-by-layer (φk=π2;φk=π4;φk=π8) ((5)–(16)) linear LB ((1), (3), (5), (7), (9), (11), (13), and (15)) and circular CB ((2), (4), (6), (8), (10), (12), (14), and (16)) birefringence **BF** theziograms. Explanation in the text.

The series of fragments in Figure 3 show the experimental results of the polarization-interference Jones matrix theziography method (relationships (7)–(20)) of linear LB(a,b) and circular CB(a,b) birefringence of **BF**:

- coordinate distributions (a×b=1000×1000 and a×b=100×100) of integral theziograms $T^{LB}\left(a,b\right)$ and $T^{CB}\left(a,b\right)$—fragments (1) and (2);
- histograms of probability distributions of N(LB(1000×1000)) and N(CB(1000×1000)) integral values of linear LB and circular LB birefringence of polycrystalline architectonics—fragments (3) and (4);
- coordinate distributions (a×b=100×100) of reconstructed (ratios (13), (14), (17), (19), and (20)) layer-by-layer theziograms $T^{LB}\left(\varphi_{k},(a,b)\right)$ (fragments (5), (9), and (13)) and $T^{CB}\left(\varphi_{k},(a,b)\right)$ (fragments (6), (10), and (14) in different phase sections (φk=π2, fragments (5) and (6)), φk=π4, (fragments (9) and (10)), φk=π8, fragments (13) and (14));
- histograms $N(LB(\varphi_{k},100\times 100))$ and $N(CB(\varphi_{k},100\times 100))$ of layer-by-layer distributions of linear and circular birefringence values of polycrystalline architectonics in different phase planes in different phase sections (φk=π2, fragments (7) and (8)), φk=π4, fragments (11) and (12)), φk=π8, fragments (15) and (16)).

Integral theziograms $T^{LB;CB}\left(a,b\right)$ were obtained by polarization-interference mapping (Figure 2) of the coordinate distributions of the elements of the Jones matrix of the blood facies (relations (7), (8), (10), (11), and (16)) with subsequent analytical reconstruction (relation (13), (14), (17), (19), and (20)) of the LB(1000×1000) and CB(1000×1000) maps.

Reconstruction (relation (17) and (19)) of the layered theziograms $T^{LB;CB}\left(\varphi_{k},100\times 100\right)$ in different phase planes (φk=π2;φk=π4;φk=π8) of the object field of complex amplitudes (relation (16)) was carried out by step-by-step phase scanning (relations (19) and (20)) and sequential algorithmic calculations (relation (13)). The analysis of the theziograms of linear $T^{LB}\left(a,b\right)$ and circular $T^{CB}\left(a,b\right)$ birefringence confirmed the adequacy of the proposed model representations (relationships (1)–(15)) about the optical anisotropy of the **BF** polycrystalline architectonics.

Comparison of coordinate (Figure 3, fragments (1), (2), (5), (6), (9), (10), (13), and (14))) and probabilistic (Figure 3, fragments (3), (4), (7), (8), (11), (12), (15), and (16))) distributions of phase anisotropy parameters (LB(1000×1000) and CB(1000×1000)) of **BF** revealed:

- individual structure of algorithmically reconstructed integral and layer-by-layer theziograms $T^{LB;CB}\left(1000\times 1000\right)$ of linear LB and circular CB birefringence of optically anisotropic architectonics—fragments (1), (3) and (2), and (4);
- dependence of the average level and the magnitude of the range of change in fluctuations of random values of phase anisotropy parameters on the magnitude of the phase parameter $\varphi_{k}$ in the reconstructed object field of complex amplitudes—fragments (5), (7), (9), (11), (13), and (15) and (6), (8), (10), (12), (14), and (16);
- the tendency to decrease the magnitude of fluctuations of random values of linear LB and circular CB birefringence in the phase planes φk=π2;φk=π4;φk=π8) of the field of complex amplitudes with smaller values of the scanning parameter $\varphi_{k}$ (relationships (19) and (20))—fragments (5), (9), and (13) and (6), (10), and (14).

The discovered features of the Jones transformation of matrix reconstructed theziograms $T^{LB;CB}\left(\varphi_{k},100\times 100\right)$ can be associated with the influence on the calculated value of the birefringence parameter of different multiplicities (q) of light scattering in the considered phase planes (φk=π2;φk=π4;φk=π8)) of the **BF** object field.

For integral theziograms $T^{LB}\left(a,b\right)$ and $T^{CB}\left(a,b\right)$ the scattering factor of laser radiation in the volume of polycrystalline blood facies is maximal. Each local (q=1) act of interactions of laser radiation with an optically anisotropic domain of polycrystalline architectonics corresponds to “true” values of the set of elements of the Jones matrix $w_{ik}$ (Equations (7) and (8)). Applying algorithm (10) to them, it is possible to reconstruct the theziograms $T^{LB;CB}\left(a,b\right)$ of the real 3D polycrystalline structure of the blood facies. This “ideal” situation is distorted (multiplied) by multiple (q) acts of interaction—scattering. As a result, the Jones algorithms for reconstructing phase anisotropy theziograms (Equation (10)) recreate coordinate distributions with significantly greater values of linear LB and circular CB birefringence. As the value of the phase scanning parameter $\varphi_{k}$ decreases (↓), components with a lower multiplicity (p<q) of light scattering are extracted from the object field of complex amplitudes. This enables Jones matrix reconstruction of the **BF** phase anisotropy theziograms $T^{LB;CB}\left(a,b\right)$ are closer to real ones.

Finally, starting from a certain level of the phase scanning parameter $\varphi_{k}$, the most informative component with a minimum (mainly single q→1) multiplicity of light scattering in the volume of the blood facies is “extracted” from the object field. In this situation, the maximum sensitivity of the reconstructed theziograms $T^{LB;CB}\left(a,b\right)$ to pathological changes in the **BF** optically anisotropic architectonics is realized. The considered scenario of layer-by-layer polarization-interference Jones matrix reconstruction of phase anisotropy parameters quantitatively characterizes the results of statistical analysis (ratios (21)) of the distributions of $LB\left(a,b\right)$ and $CB\left(a,b\right)$—Table 2.

The following has been established:

- individual and different Gaussian ($Z_{3;4}\left(LB,CB\right)=0$) statistics of holographically reproduced **BF** theziograms LB(a,b) and $CB\left(a,b\right)—Z_{i=1;2;3;4}\left(LB,CB\right)\neq 0$;
- the third and fourth orders of statistical moments $Z_{i=3;4}\left(LB,\;CB(a,b)\right)$ were the most sensitive to changes in the topographic structure of **BF** phase anisotropy theziograms;
- twofold range of phase $\varphi_{k}$ change (for different conditions of light scattering in the BF volume) of $Z_{i=3;4}\left(LB(a,b)\right)$ and $Z_{i=3;4}\left(CB(a,b)\right)$ values.

The results obtained may be related to the following physical considerations, which are based on the model analysis ((1)–(20)) of the **BF** optical-anisotropic properties.

We have already noted that the biochemical structure of blood can be conditionally represented in the form of a “two-component mixture”—plasma and shaped elements.

The magnitude of linear birefringence LB is mainly determined by the degree of needle crystals optical axes spatial orientation (γ) of the marginal albumin layer, which can be quite large—0≤γ≤2π [9,10,57,58,59]. As a result, the first and second order statistical moments, which characterize the mean and variance of the distributions LB(a,b), have a small value. The values of higher orders statistical moments $Z_{i=3;4}\left(LB(a,b)\right)$, which determine the asymmetry and excess of polycrystalline dendritic network BF, are quite large—Table 2.

The magnitude of the **BF** circular birefringence (globulin crystals and shaped elements of erythrocytes, platelets, leukocytes, etc.) [15,16,57,58,59], is quite large. Moreover, for a drop of blood, a significant concentration of spherulites provides an almost commensurate value for the entire volume of the optical activity parameter with the value of the linear birefringence parameter LB of the needle dendritic network of albumin crystals. Accordingly, the statistical moments $Z_{i=1;2}\left(CB\right)\approx Z_{i=1;2}\left(LB\right)$ and $Z_{i=3;4}\left(CB\right)\approx Z_{i=3;4}\left(LB\right)$ are given in Table 2.

In digital holographic phase scanning of volumetric depth **BF**, various implementations of polycrystalline networks with optically anisotropic parameter distributions (directions of optical axes, phase shifts between orthogonal amplitude components) are consistently distinguished. As a result, individual orientation distributions ($\gamma_{min}\leq \gamma \leq \gamma_{max}$) with a certain random variance of their values are reproduced for each phase plane. In addition, when the conditions of small or single scattering are reached, the range of changes in the values of random phase shifts narrows. Approaching the conditions of an optically thin layer (ratios (1)–(14)), the statistics of the **BF** phase anisotropy (LB(a,b) and CB(a,b)) changes in the following scenarios—$\varphi_{k}↓\;⟹\left(\begin{array}{c} Z_{1;2}(LB,CB)↓;\\ Z_{3;4}(LB,CB)↑ \end{array}\right)$.

Along with the phase-shifting capacity of the supramolecular networks of blood facies, mechanisms of optically anisotropic absorption are realized, the reconstruction and analysis of which is devoted to the next part of the article.

### 4.4. Jones Matrix Theziography of Amplitude Anisotropy of Polycrystalline Blood Films

**Figure 4:**
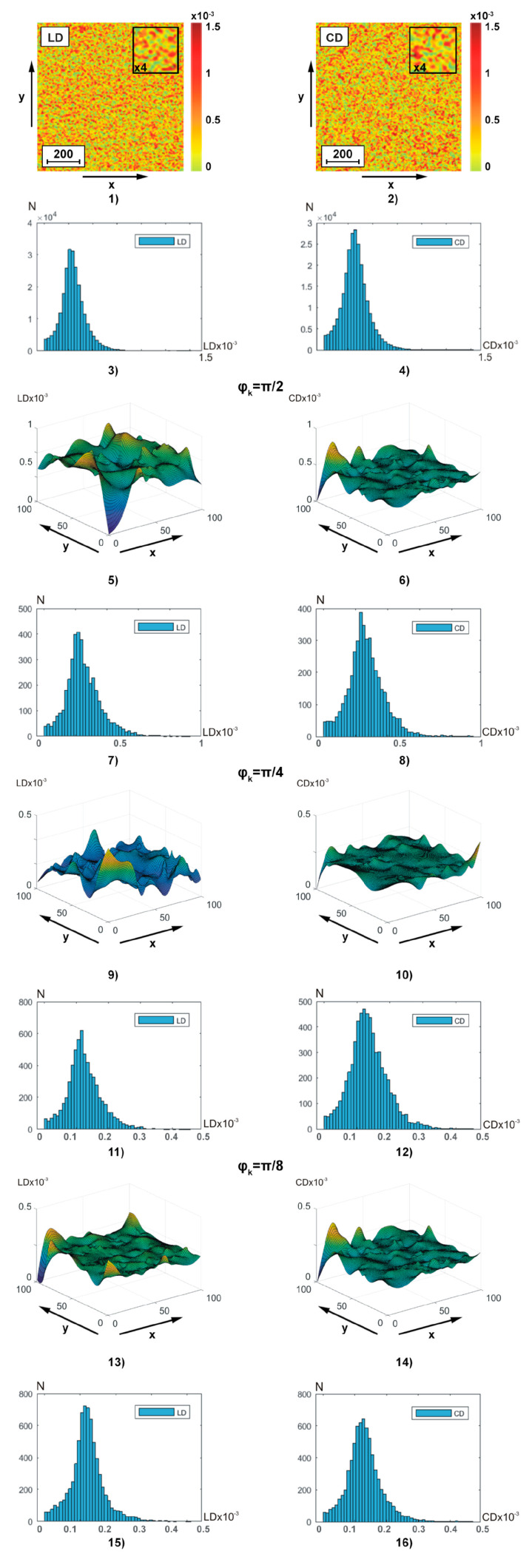
Two-dimensional distributions and histograms of integral ((1)–(4)) and layer-by-layer (φk=π2;φk=π4;φk=π8) ((5)–(16)) linear LD ((1), (3), (5), (7), (9), (11), (13), (15)) and circular CD ((1), (3), (5), (7), (9), (11), (13), (15)) birefringence **BF** theziograms. Explanation in the text.

Figure 4 illustrates, similar to the data of polarization-interference Jones matrix phase theziography (Figure 2), coordinate (fragments (1), (2), (5), (6), (9), (10), (13), and (14)) and probability (fragments (3), (4), (7), (8), (11), (12), (15), and (16)) distributions of linear LD (fragments (1), (3), (5), (7), (9), (11), (13), and (15)) and circular CD (fragments (2), (4), (6), (8), (10), (12), (14), and (16)) dichroism of **BF** polycrystalline architectonics. Integral theziograms $T^{LD}\left(a,b\right)$ and $T^{CD}\left(a,b\right)$ of amplitude anisotropy were reconstructed using algorithms (7), (8), and (11). To recreate layer-by-layer theziograms $T^{LD;CD}\left(\varphi_{k},a,b\right)$ of linear LD and circular CD dichroism, relations (14), (18), (19), and (20) were used.

From the analysis of the obtained theziograms $T^{LD;CD}\left(a,b\right)$ and $T^{LD;CD}\left(\varphi_{k},a,b\right)$ of amplitude anisotropy (Figure 4) it is evident that for each coordinate distribution of random values of the magnitude of linear and circular dichroism of the **BF** polycrystalline architectonics, an individual coordinate (fragments (1), (5), (9), and (13) and fragments (2), (6), (10), and (14)) and probabilistic (fragments (3), (7), (11), and (15) and fragments (4), (8), (12), and (16)) structure is characteristic. A comparison of a series of layered theziograms $T^{LD}\left(\varphi_{k},100\times 100\right)$ of amplitude anisotropy (Figure 4, fragments (5), (7), (9), (11), (13), and (15)) and $T^{CD}\left(\varphi_{k},100\times 100\right)$ (Figure 4, fragments (6), (8), (10), (12), (14), and (16)) demonstrated a tendency, similar to the phase theziograms $T^{LB}\left(\varphi_{k},100\times 100\right)$ and $T^{CB}\left(\varphi_{k},100\times 100\right)$ (Figure 4), of decreasing the magnitude of fluctuations of the parameters of optically anisotropic absorption in the phase planes (φk=π2;φk=π4;φk=π8),respectively) of the components of the object field of complex amplitudes with a low multiplicity of light scattering.

Quantitively phase transformation of the LD(a,b) and CD(a,b) theziograms is illustrated the 1st—4th orders central statistical moments—Table 3.

Analysis has shown that:

- difference from zero (for all phase facies depths) values of all central statistical moment’s $Z_{i=1;2;3;4}\left(LD,CD\right)\neq 0$;
- dependence of the magnitude of statistical parameters $Z_{i=1;2;3;4}\left(LD,CD\right)↔q\left(\varphi_{k}\right)$ from the phase scan $\varphi_{k}$ depth;
- the most sensitive markers for changes in amplitude anisotropy theziograms are 3rd and 4th orders statistical moments $Z_{i=3;4}\left(LD,CD,\left(a,b\right)\right)$;
- as the phase scanning parameter $\varphi_{k}↑$ value increases (approaching single scattering conditions), the statistical parameters $Z_{i=3;4}\left(LD,\left(a,b\right)\right)$ values increase to 85%, and $Z_{i=3;4}\left(CD,\left(a,b\right)\right)$ increase by 60–80%.

From a physical point of view, the obtained results can be related to the fact that the value of linear dichroism LD, as well as linear birefringence, at all phase depths, is determined by the degree of spatial orientation optical axes needle protein crystals (albumin, fibrin, and elastin). Under the conditions of phase approximation to the optically thin layer, the changes in the orientations of the needle crystals optical axes, as well as the magnitude of phase shifts, the following scenario of changes in the first and fourth orders statistical moments magnitude is implemented—$\varphi_{k}↓\;⟹\left(\begin{array}{c} Z_{1;2}(LD,CD)↓;\\ Z_{3;4}(LD,CD)↑ \end{array}\right)$—Table 3. Along with this, the value of circular dichroism CD combined “shaped elements—albumin—globulin grids” polycrystalline network (globulin crystals, shaped elements) is not less than the value of linear dichroism LD. Accordingly, the statistical moments $Z_{i=1;2}\left(LD\right)\approx Z_{i=1;2}\left(CD\right)$ and $Z_{i=3;4}\left(LD\right)\approx Z_{i=3;4}\left(CD\right)$. Thus, the conducted cycle of fundamental polarization-interference studies **BF** optical anisotropy polycrystalline structure layer-by-layer maps is provided the basis for the applied Jones matrix theziography implementation.

The most sensitive markers ($Z_{3;4}(LB,CB,LD,CD)$) to changes in the optical anisotropy of the BF supramolecular networks, determined within the framework of statistical analysis of layered Jones matrix theziograms of the blood facies supramolecular networks, were used in applied studies of the diagnostic power of the polarization-interference method in the early detection of papillary thyroid cancer and differentiation of its stages.

## 5. Diagnostic Capabilities of Jones Matrix Theziography of Polycrystalline Blood Films

This part of the work includes three data arrays of experimental studies of blood facies.

The first part is devoted to a comparative study of the statistical structure of Jones matrix theziograms of linear and circular birefringence and dichroism of blood facies of donors (control group 1, 26 samples) and patients with the first stage of papillary cancer (stage 1—experimental group 2, 26 samples).

The second part is devoted to the study the diagnostic efficiency by **BF** layer-by-layer Jones matrix theziography for detection early stage of papillary thyroid carcinoma.

The third part contains the results of a comparative analysis of the effectiveness of diagnostics (group 1) and differentiation of stage 1 (group 2) and stage 2 (group 3, 26 samples) thyroid cancer using Jones matrix theziography and Mueller matrix tomography of blood facies.

### 5.1. Jones Matrix Theziograms of BF Phase and Amplitude Anisotropy

The diagnostic methodology of the developed polarization-interference method includes the following stages:

- polarization-interference mapping (Figure 2) of object fields of the set of samples of blood facies of group 1, group 2 and group 3;
- calculation (ratios (7), (8)) of the integral elements of the Jones matrix and the matrix operator of “single” (phase plane φk=π8) scattering;
- reconstruction of integral $T^{LB,CB,LD,CD}\left(a,b\right)$ and layer-by-layer TLB,CB,LD,CDφk=π8,a,b theziograms of the **BF** phase and amplitude anisotropy;
- statistical analysis (ratios (21)) of theziograms and determination of the most sensitive parameters (markers) $Z_{i}\left(LB,CB.LD,CD\right)$ and Ziφk=π8,LB,CB.LD,CD to pathological changes in the **BF** optically anisotropic architecture;

#### 5.1.1. Phase Anisotropy Theziograms $T^{LB,CB}\left(a,b\right)$ and TLB,CBφk=π8,a,b

**Figure 5:**
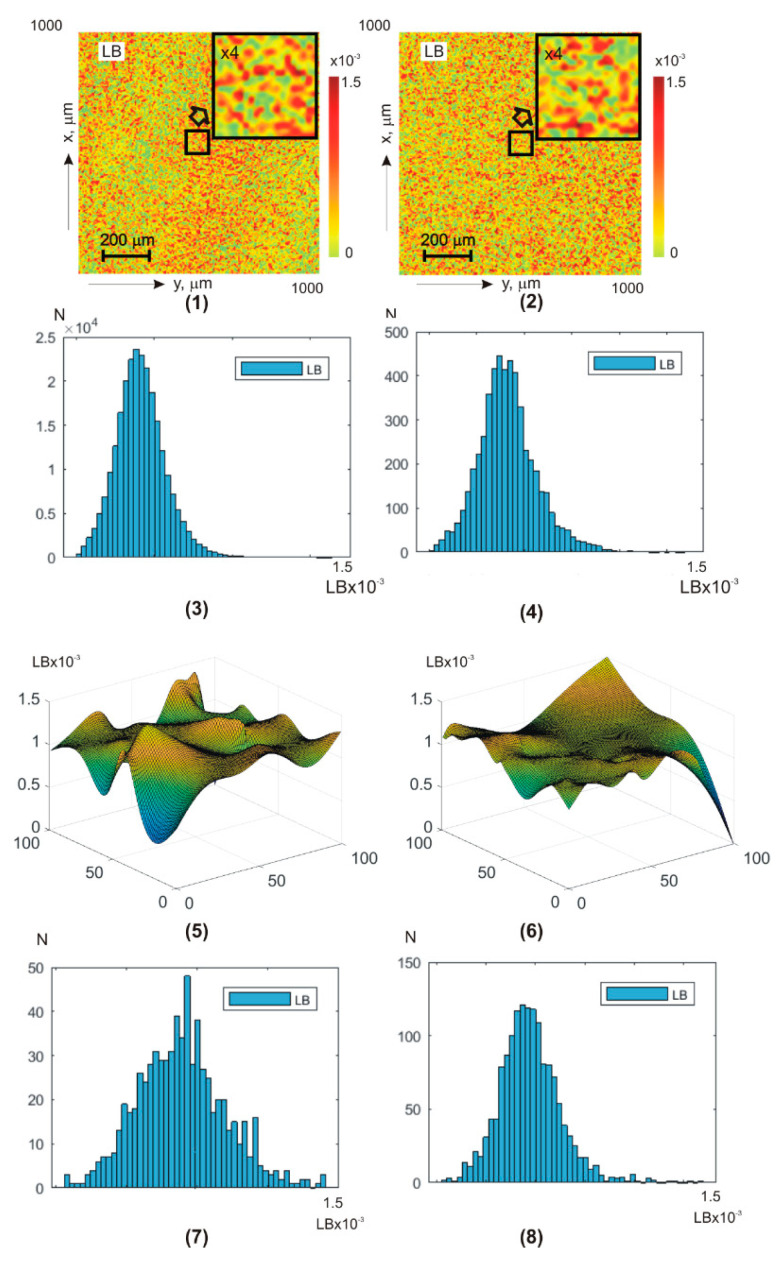
**BF** theziograms {LB(a,b)} and histograms G{LB(a,b)} of healthy donors (left column) and patients with papillary thyroid carcinoma (right column). Explanation in the text.

**Figure 6:**
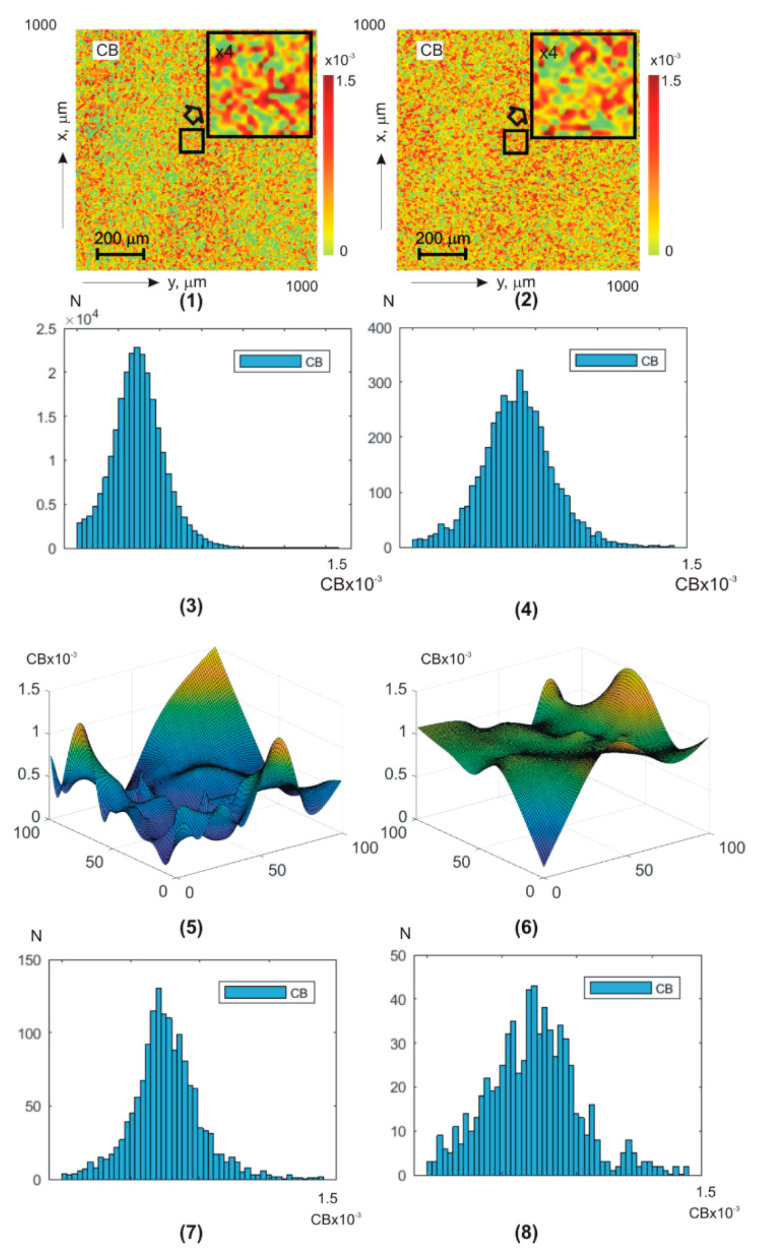
**BF** theziograms {CB(a,b)} and histograms G{CB(a,b)} of healthy donors (left column) and patients with papillary thyroid carcinoma (right column). Explanation in the text.

Figure 5 and Figure 6 show integral (a,b) (fragments (1) and (2)) and layer-by-layer $\left(\varphi_{k},\left(a,b\right)\right)$ (fragments (5) and (6)) linear $LB\left(\varphi_{k},\left(a,b\right)\right)$ (Figure 4) and circular $CB\left(\varphi_{k},\left(a,b\right)\right)$ (Figure 5) birefringence theziograms, as well as histograms of G(LB,CB) distributions (fragments (3), (4), (7), and (8)) from control group 1 (fragments (1), (3), (5), and (7)) and experimental group 2 (fragments (2), (4), (6), and (8)).

Quantitative phase transformation and intergroup differences between the $LB\left(\varphi_{k},\left(a,b\right)\right)\;$and $CB\left(\varphi_{k},\left(a,b\right)\right)$ **BF** theziograms are illustrated by the statistical analysis results—Table 4.

Statistical analysis of the phase anisotropy theziograms LB(a,b) and CB (a,b) revealed:

- for all phase depths, the central first–fourth-order statistical moments are non-zero-$Z_{i=1;2;3;4}\left(LB,CB\right)\neq 0$ the third and fourth order statistical moments $Z_{i=3;4}\left(LB(a,b),CB(a,b)\right)$ are the most sensitive markers (especially in the phase plane $\varphi_{k}=\pi /8$) to changes in **BF** birefringence;
- for theziograms $LB\left(\varphi_{k}=\pi /8;(a,b)\right)$, the differences ${∆Z}_{i=3;4}\left(LB(a,b)\right)$ between the values of BF control and experimental groups diagnostic markers are 30–40%;
- for theziograms $CB\left(\varphi_{k}=\pi /8;(a,b)\right)$, the differences ${∆Z}_{i=3;4}\left(CB(a,b)\right)$ between the values of diagnostic markers increase and reach 45–55%.

From a physical point of view, the results obtained can be associated with the following features of **BF** architectonics polycrystalline networks pathological changes.

First, in previous classical vector parametric studies of optical anisotropy blood films and their plasma was demonstrated that the linear birefringence LB value is mainly determined by the degree of the albumin, fibrin, and elastin needle crystals’ optical axes spatial orientation [54,55,56].

The orientation changes in the directions of the optical axes in the dendritic grid are very large and almost equidistant—0≤γ≤2π. As a result, the values of the first and second order statistical moments, which characterize $LB\left(a,b\right)\;$distributions’ means and variance, are quite large. The values of higher-order statistical moments $Z_{i=3;4}\left(LB(a,b)\right)$, on the contrary, are insignificant.

During the cancer process, blood crystallization increases. Due to this, for patients’ blood droplets, dehydrated films from experimental group 2, the range of changes decreases. As a result, the parameters $Z_{i=1;2}\left(LB(a,b)\right)↓$ decrease and, conversely, the third and fourth order $Z_{i=3;4}\left(LB(a,b)\right)↑\;$ statistical moments increase.

Secondly, the value of the globulin crystals and shaped elements (erythrocytes, platelets, leukocytes, and monocytes) of the spherulite optically anisotropic network of the circular birefringence CB [53,54,55] is commensurate with the linear birefringence LB values. With cancer inflammation, the globulin and white blood cell molecule concentration increases. This increases the blood droplets’ dehydrated films’ chiral molecular domains’ optical activity in patients with papillary thyroid carcinoma samples.

By this, first–fourth-order statistical moments, which characterize the circular birefringence random values distributions, undergo the following changes$:\;Z_{i=1;2}\left(CB\right)↑\;$and, conversely, $Z_{i=3;4}\left(CB\right)↓$.

Third, as the scan $\varphi_{k}↓$ phase shift decreases, a more optically thin rat blood-dehydrated film volume is released.

As a result, the albumin, fibrin, and elastin needle crystals’ optical axes directions distribution are transformed into an asymmetric one ($\gamma_{min}\leq \gamma \leq \gamma_{max}$) with a value certain random dispersion. In addition, the range of changes in random values of phase shifts between laser radiation amplitudes orthogonal components is narrowed. These changes in the phase anisotropy theziograms statistics of dehydrated blood drop films LB(a,b) and $CB\left(a,b\right)—\left(\begin{array}{c} Z_{1;2}(LB,CB)↓;\\ Z_{3;4}(LB,CB)↑ \end{array}\right)$. Against this background, cancer differences in the optically anisotropic network’s polycrystalline structure of blood samples from experimental group 2 are more clearly revealed.

#### 5.1.2. Amplitude Anisotropy Theziograms $T^{LD,CD}\left(a,b\right)$ and TLD,CDφk=π8,a,b

**Figure 7:**
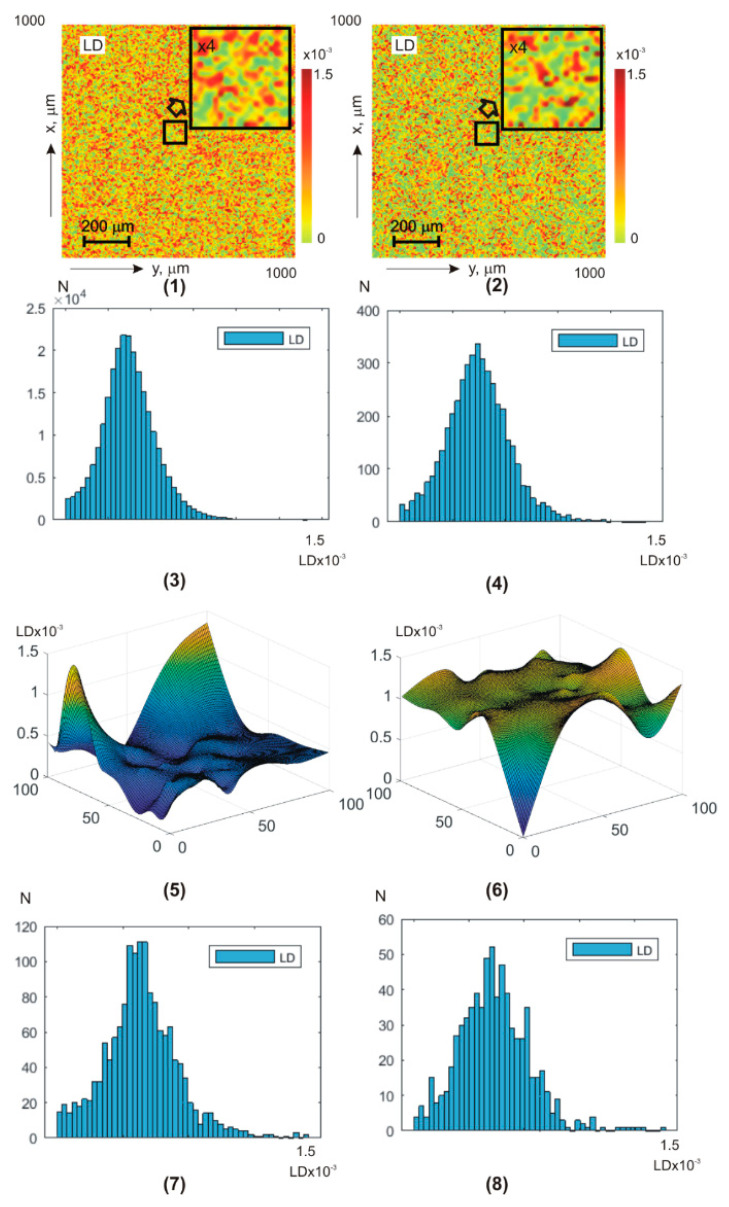
**BF** LD theziograms {LD(a,b)} and histograms G{LD(a,b)} of healthy donors (left column) and patients with papillary thyroid carcinoma (right column). Explanation in the text.

**Figure 8:**
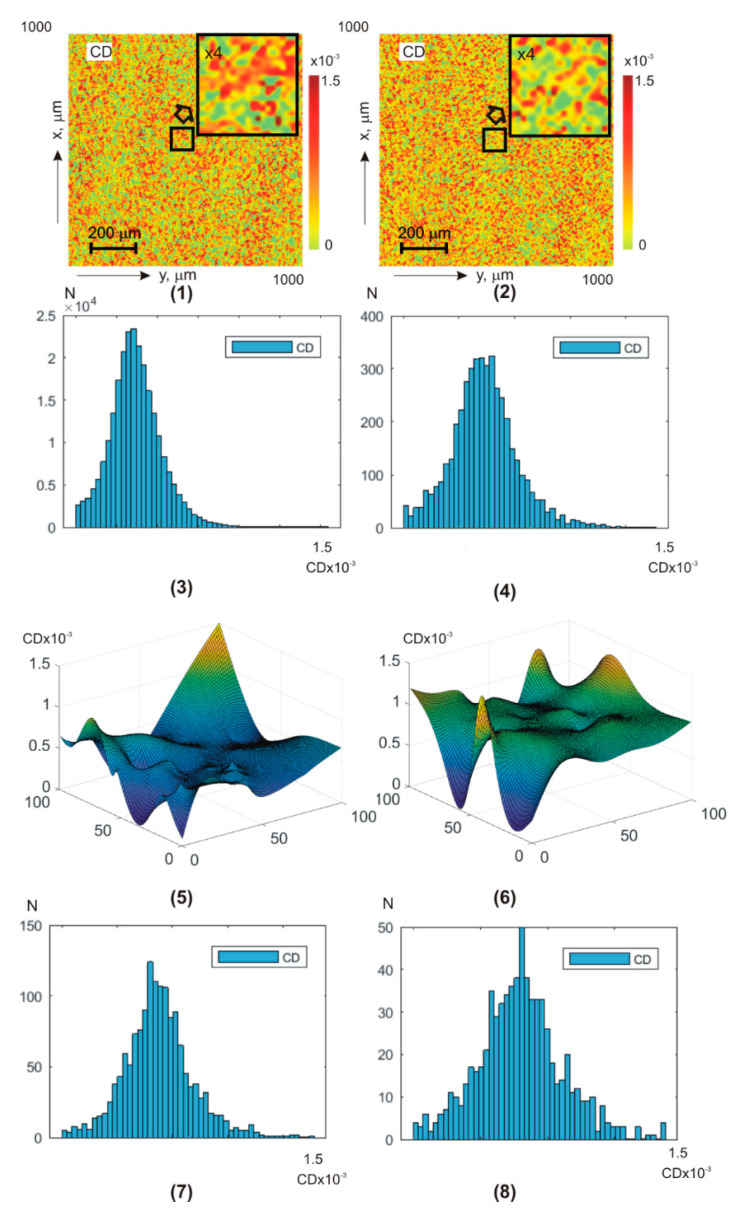
**BF** theziograms {CD(a,b)} and histograms G{CD(a,b)} of healthy donors (left column) and patients with papillary thyroid carcinoma (right column). Explanation in the text.

Figure 7 and Figure 8 show integral (a,b) (fragments (1) and (2)) and layer-by-layer $\left(\varphi_{k},(a,b)\right)$ (fragments (5), (6)) $LD\left(\varphi_{k},(a,b)\right)$ (Figure 7) and $CD\left(\varphi_{k},(a,b)\right)$ (Figure 8) theziograms, as well as histograms of G(LD,CD) (fragments (3), (4), (7), and (8)).

Control group 1 (fragments (1), (3), (5), and (7)) and experimental group 2 (fragments (2), (4), (6), and (8)).

Analysis of **BF** amplitude anisotropy theziograms for all phase depths revealed:

- difference all first–fourth-order statistical moment values from zero $Z_{i=1;2;3;4}\left(LD,CD\right)\neq 0;$ dependence of statistical distributions $Z_{i=1;2;3;4}\left(LD,CD\right)↔q\left(\varphi_{k}\right)\;$on the value of the phase scanning parameter $\varphi_{k}\;$of **BF** polycrystalline architectonics;
- the most sensitive diagnostic markers are third- and fourth-order statistical moments $Z_{i=3;4}\left(LD(a,b),CD(a,b)\right)$;
- for linear dichroism $LD\left(a,b\right)\;$theziograms, the differences ${∆Z}_{i=3;4}\left(LD(a,b)\right)\;$between the diagnostic marker’s values of the control and experimental **BF** samples groups are 25–30%;
- for $CD\left(a,b\right)$ circular dichroism theziograms, the differences ${∆Z}_{i=3;4}\left(CD(a,b)\right)$ between the diagnostic marker’s values increase and reach 35–45%.

Quantitative phase transformation and intergroup differences between the $LD\left(\varphi_{k},\left(a,b\right)\right)$ and $CD\left(\varphi_{k},\left(a,b\right)\right)\;$**BF** theziograms are illustrated by the statistical analysis results—Table 5.

From a physical point of view, the results obtained can be attributed to the fact that phase scanning $\varphi_{k}↓$ provides the ability to isolate a more optically thin volume of **BF**. Due to this, the topographic structure of **BF** theziograms becomes as close as possible to the statistics of the needle crystals (linear dichroism) optical axes directions distribution, as well as the values of phase shifts (circular dichroism). As a result, the maximum possible accuracy of detecting polycrystalline differences between **BF** rat samples from both groups is achieved.

A slightly lower accuracy level (information analysis of phase anisotropy theziograms—Table 5) can be associated with a lower level of anisotropic absorption compared to laser scattering by **BF** samples. As a result, the sensitivity of the **BF** polycrystalline component matrix mapping decreases.

It should be noted that the sensitivity of the developed and experimentally tested method of polarization-interference Jones matrix theziography of blood facies in the diagnosis of early stages of papillary thyroid cancer can be improved by further improving the algorithmic apparatus to eliminate the influence of the depolarized background during the reconstruction of the anisotropy parameters of polycrystalline architecture.

### 5.2. Information Analysis of the Diagnostic Efficiency of Matrix Polarization-Interference Methods

The information analysis of the experimental results uses a set of operational characteristics from evidence-based medicine [60,61]:

- Sensitivity (Se)—is the proportion of true positive results (N=Q−n) of the diagnostic method among all samples in the experimental group 2 (Q); n—false negative results
  (22) $$Se=\frac{N}{Q}100\%$$
- Specificity (Sp)—is the proportion of true negative results (H=G−h) of the method among all samples in the control group 1 (G); h—false positive results
  (23) $$Sp=\frac{H}{G}100\%$$
- Accuracy (Ac)—is the proportion of correct results (A+B) of the test among all samples (N+H)
  (24) $$Ac=\frac{A+B}{N+H}100\%$$
  If (Q=G), then Ac is referred to as balanced accuracy.

To assess the levels of diagnostic accuracy and differentiation of changes in the parameters of optical anisotropy of polycrystalline films of blood facies, we used the classification given in [61]—Table 6. This provides the possibility of conducting a comparative analysis of the diagnostic efficiency of the polarization-interference Jones matrix theziography method with the data of 3D Mueller matrix studies of pathological and necrotic changes in the optical anisotropy of biological preparations [34,35].

### 5.3. Comparative Information Analysis of Jones Matrix Theziography and Mueller Matrix Tomography Data

This section presents the results of a comparative analysis with a group of donors (group 1) of the diagnostic power of Jones matrix theziography and Mueller matrix tomography of blood facies in early diagnostics of stage 1 (the formation develops inside the gland; the organ capsule is not deformed and symptoms do not appear, group 2); detection of stage 2 (proliferation of nodes and deformation of the capsule, groups 3–26 samples) of thyroid cancer and differentiation of cancer stages (group 2 and group 3). The studies were conducted in the most sensitive phase plane φ=π⁄8, Table 7.

The following maximum levels of balanced accuracy for papillary thyroid cancer diagnostics have been established.

1. Jones matrix theziography (stage 1):
  - linear birefringence LB(a,b)—good level 88.5% (Se=88.5%;Sp=88.5%);
  - circular birefringence CB(a,b)—very good level 90.4% (Se=92.3%;Sp=88.5%);
  - linear dichroism LD(a,b)—good level 82.7% (Se=84.6%;Sp=80.8%);
  - circular dichroism CD(a,b)—good level 88.5% (Se=88.5%;Sp=88.5%).
2. Mueller matrix tomography (stage 1)—unsatisfactory accuracy Ac<80% for tomograms of linear and circular birefringence and dichroism.
3. Jones matrix theziography (stage 2):
  - linear birefringence LB(a,b)—very good level 94.2% (Se=96.2%;Sp=88.5%);
  - circular birefringence CB(a,b)—excellent level 96.2% (Se=96.2%;Sp=96.2%);
  - linear dichroism LD(a,b)—very good level 92.3% (Se=96.2%;Sp=88.5%);
  - circular dichroism CD(a,b)—very good level 94.2% (Se=92.3%;Sp=96.2%).
4. Mueller matrix tomography (stage 2):
  - linear birefringence LB(a,b)—good level 86.5% (Se=88.5%;Sp=84.6%);
  - circular birefringence CB(a,b)—good level 88.2% (Se=92.3%;Sp=84.6%);
  - linear LD(a,b) and circular dichroism CD(a,b)—unsatisfactory level (Se;Sp;Ac<80%).

The following maximum levels of balanced accuracy of differentiation of papillary thyroid cancer stages were established.

1. Jones matrix theziography (stages 2 and 3):
  - linear birefringence LB(a,b)—very good level 92.3% (Se=92.3%;Sp=92.3%);
  - circular birefringence CB(a,b)—very good level 94.2% (Se=96.2%;Sp=92.3%);
  - linear dichroism LD(a,b)—very good level 90.4% (Se=88.5%;Sp=92.3%);
  - circular dichroism CD(a,b)—very good level 92.3% (Se=92.3%;Sp=92.3%).
2. Mueller matrix diffuse tomography (stages 2 and 3):
  - linear birefringence LB(a,b)—good level 82.7% (Se=80.8%;Sp=84.6%);
  - circular birefringence CB(a,b)—good level 84.6% (Se=88.5%;Sp=80.8%);
  - linear LD(a,b) and circular dichroism CD(a,b)—unsatisfactory level (Se;Sp;Ac<80%).

## 6. Prospects for Further Research

In order to expand the functionality and increase the sensitivity of the developed technique of laser polarization-interference theziography of blood facies, the following set of theoretical and experimental studies will be implemented:

- Modernization of the algorithmic description of the processes of formation of object fields of complex amplitudes by using the logarithmic decomposition [62,63,64,65] of the Jones matrix on the basis of differential matrices of the first (single scattering) and second (diffuse component) components. This will ensure the possibility of almost complete elimination of the influence of the depolarized background and will maximally increase the sensitivity, specificity, and accuracy of differential diagnostics cancer stages.
- The developed technique of polarization-interference Jones matrix differential theziography of blood facies will be tested in diagnostics of various benign and malignant pathologies of the thyroid gland—nodular goiter, autoimmune thyroiditis, and papillary cancer of stages 1–4.
- Diagnostically relevant relationships between Jones matrix theziograms and the features of the polycrystalline architectonics of the facies of other types of biological fluids—saliva (laryngeal cancer), bile (cholecystitis, cholelithiasis), urine (albuminuria), and synovial fluid (joint inflammation) will be determined and studied.
- In order to increase the reliability of the data obtained using the polarization-interference Jones matrix theziography method, additional diagnostic markers will be developed within the framework of correlation (statistical moments that characterize the distribution of autocorrelation functions), wavelet (distribution of the amplitudes of the wavelet coefficients of optical anisotropy maps), and fractal (spectra of singularities of the distributions of optical anisotropy parameters) analysis.

## 7. Conclusions

The main fundamental result of the conducted research is a new polarization-interference multifunctional technique of laser Jones matrix theziography of the polycrystalline structure of supramolecular networks of dehydrated blood films was developed. This method increased the sensitivity and expanded the functional capabilities of Mueller matrix tomography, which provided the possibility of minimally invasive early diagnosis of asymptomatic stage 1 papillary cancer, as well as differentiation of its first and second stages.

An equally important, “instrumental” result is the possibility of a more express (four dimensions) in comparison with Mueller matrix (eight dimensions) direct polarization-interference extraction of Jones matrix images from phase-stationary speckle fields.

As a result, a set of blood facies polycrystalline component phase (birefringence—LB,CB) and amplitude (dichroism—LD,CD) anisotropy integral and layered maps (a,b) was obtained and physically analyzed.

Regularities and statistical scenarios of phase transformations in the structure of holographically reconstructed theziograms of optical anisotropy of blood facies have been established—$\left(\varphi_{k}↓\right)⟹\left(\begin{array}{c} Z_{i=1;2}\left(LB,CB,LD,CD(a,b)\right)↓;\\ Z_{i=3;4}\left(LB,CB,LD,CD(a,b)\right)↑ \end{array}\right)$.

The most sensitive markers to changes in the layer–by–layer topographic structure of blood facies phase and amplitude anisotropy are established—the third- and fourth-order $Z_{i=3;4}\left(LB,CB,LD,CD(a,b)\right)$ statistical moments.

The main advantages of the Jones matrix method in comparison with the Mueller matrix diffuse tomography technique are shown, as follows:

- high accuracy of early diagnostics (stage 1: JM−90.4% and MMT−78.8%) of papillary cancer at its asymptomatic stage;
- excellent detection rate of stage 2 papillary cancer: JM−96.2% and MMT−88.5%) of cancer diagnostics
- very good accuracy (JM−94.2% and MMT−82.7%) of differentiation of papillary thyroid cancer stages.

## Figures and Tables

**Table 1:** **BF** samples optical parameters.

Parameters	BF
$\mathrm{Attenuation}\;(\mathrm{extinction})\;\mathrm{coefficient}\;\tau ,cm^{-1}$	0.38 ± 0.021
Depolarization degree Λ,%	29 ± 0.18

**Table 2:** First–fourth-order statistical moments that characterize the LB(a,b) and CB(a,b) theziograms.

$Z_{i}$	LB	LB(φ=π/2)	LB(φ=π/4)	LB(φ=π/8)
$Z_{1}\times 10^{-3}$	0.42 ± 0.082	0.27 ± 0.071	0.16 ± 0.053	0.093 ± 0.0048
$Z_{2}\times 10^{-3}$	0.31 ± 0.016	0.28 ± 0.015	0.24 ± 0.014	0.21 ± 0.012
$Z_{3}$	0.35 ± 0.018	0.41 ± 0.023	0.53 ± 0.027	0.65 ± 0.036
$Z_{4}$	0.51 ± 0.028	0.67 ± 0.037	0.78 ± 0.042	0.91 ± 0.046
$Z_{i}$	CB	CB(φ=π/2)	CB(φ=π/4)	CB(φ=π/8)
$Z_{1}\times 10^{-3}$	0.32 ± 0.067	0.13 ± 0.055	0.094 ± 0.0046	0.089 ± 0.0045
$Z_{2}\times 10^{-3}$	0.29 ± 0.017	0.23 ± 0.013	0.19 ± 0.011	0.17 ± 0.009
$Z_{3}$	0.56 ± 0.029	0.71 ± 0.037	0.82 ± 0.043	0.99 ± 0.047
$Z_{4}$	0.71 ± 0.038	0.94 ± 0.046	1.17 ± 0.064	1.38 ± 0.073

**Table 3:** 1st–4th orders statistical moments of the **BF** linear and circular dichroism.

$Z_{i}$	LD	LD(φ=π/2)	LD(φ=π/4)	LD(φ=π/8)
$Z_{1}\times 10^{-3}$	0.24 ± 0.069	0.19±0.064	0.07 ± 0.058	0.06 ± 0.054
$Z_{2}\times 10^{-3}$	0.28 ± 0.015	0.24 ± 0.013	0.22 ± 0.013	0.18 ± 0.009
$Z_{3}$	0.41 ± 0.022	0.56 ± 0.029	0.68 ± 0.036	0.77 ± 0.039
$Z_{4}$	0.62 ± 0.033	0.79 ± 0.041	0.92 ± 0.046	0.09 ± 0.051
$Z_{i}$	CD	CD(φ=π/2)	CD(φ=π/4)	CD(φ=π/8)
$Z_{1}\times 10^{-3}$	0.095 ± 0.0055	0.093 ± 0.0049	0.088 ± 0.0046	0.082 ± 0.0045
$Z_{2}\times 10^{-3}$	0.21 ± 0.011	0.19 ± 0.011	0.17 ± 0.009	0.15 ± 0.008
$Z_{3}$	0.65 ± 0.034	0.81 ± 0.043	0.97 ± 0.051	0.16 ± 0.066
$Z_{4}$	0.83 ± 0.043	0.05 ± 0.054	0.31 ± 0.072	0.54 ± 0.087

**Table 4:** Statistical parameters of $LB\left(\varphi_{k},(a,b)\right)$ and $CB\left(\varphi_{k},(a,b)\right)$.

$Z_{i}$	LB		CB	
	Group 1	Group 2	Group 1	Group 2
$Z_{1}\times 10^{-3}$	0.41 ± 0.081	0.34 ± 0.081	0.02 ± 0.062	0.11 ± 0.064
$Z_{2}\times 10^{-3}$	0.33 ± 0.015	0.28 ± 0.015	0.27 ± 0.015	0.32 ± 0.016
$Z_{3}$	0.31 ± 0.017	0.42 ± 0.021	0.59 ± 0.025	0.49 ± 0.027
$Z_{4}$	0.45 ± 0.026	0.53 ± 0.027	0.75 ± 0.042	0.64 ± 0.036
$Z_{i}$	$\left\{LB\left(\pi /8\right)\right\}$		$\left\{CB\left(\pi /8\right)\right\}$	
	Group 1	Group 2	Group 1	Group 2
$Z_{1}\times 10^{-3}$	0.89 ± 0.046	0.94 ± 0.049	0.88 ± 0.049	0.95 ± 0.046
$Z_{2}\times 10^{-3}$	0.21 ± 0.011	0.24 ± 0.013	0.16 ± 0.008	0.21 ± 0.011
$Z_{3}$	0.68 ± 0.035	0.57 ± 0.037	0.69 ± 0.039	0.87 ± 0.048
$Z_{4}$	0.89 ± 0.044	0.81 ± 0.048	0.82 ± 0.044	1.07 ± 0.059

**Table 5:** Statistical parameters of $LD\left(\varphi_{k},(a,b)\right)$ and $CD\left(\varphi_{k},(a,b)\right)$.

$Z_{i}$	LD		CD	
	Group 1	Group 2	Group 1	Group 2
$Z_{1}\times 10^{-3}$	0.21 ± 0.063	0.29 ± 0.068	0.89 ± 0.051	0.94 ± 0.054
$Z_{2}\times 10^{-3}$	0.27 ± 0.014	0.33 ± 0.016	0.18 ± 0.011	0.21 ± 0.011
$Z_{3}$	0.51 ± 0.023	0.43 ± 0.023	0.76 ± 0.036	0.63 ± 0.036
$Z_{4}$	0.76 ± 0.032	0.64 ± 0.034	0.93 ± 0.047	0.81 ± 0.044
$Z_{i}$	$\left\{LD\left(\pi /8\right)\right\}$		$\left\{CD\left(\pi /8\right)\right\}$	
	Group 1	Group 2	Group 1	Group 2
$Z_{1}\times 10^{-3}$	0.94 ± 0.054	0.89 ± 0.051	0.81 ± 0.046	0.88 ± 0.043
$Z_{2}\times 10^{-3}$	0.18 ± 0.009	0.15 ± 0.008	0.14 ± 0.007	0.18 ± 0.009
$Z_{3}$	0.79 ± 0.038	0.91 ± 0.045	1.09 ± 0.068	0.91 ± 0.067
$Z_{4}$	0.98 ± 0.052	1.21 ± 0.062	1.44 ± 0.089	1.15 ± 0.089

**Table 6:** Threshold balanced accuracy levels.

Diagnostic Accuracy Assessment	Accuracy, Ac,%
Unsatisfactory	<80
Satisfactory	81–85
Good	86–90
Very good	91–95
Excellent	>95

**Table 7:** Operational characteristics of the diagnostic power of the Jones matrix theziography and Mueller matrix tomography of blood facies methods.

Z3;4π8	$LB\left(a,b\right)$					
Groups	**“1”**	**“2”**	**“1”**	**“3”**	**“2”**	**“3”**
$Se\left(JT\right),\%$	N=23;n=3; **88.5**		N=25 ;n=1; **96.2**		N=24 ;n=2; **92.3**	
$Se\left(MMT\right),\%$	N=20 ;n=6; **76.9**		N=23 ;n=3; **88.5**		N=21;n=5; **80.8**	
$Sp\left(JT\right),\%$	H=23;h=3; **88.5**		H=24 ;h=2; **88.5**		H=24 ;h=2; **92.3**	
$Sp\left(MMT\right),\%$	H=20 ;h=6; **76.9**		H=22 ;h=4; **84.6**		H=22 ;h=4; **84.6**	
$Ac\left(JT\right),\%$	**88.5**		**94.2**		**92.3**	
$Ac\left(MMT\right),\%$	**76.9**		**86.5**		**82.7**	
Z3;4π8	$CB\left(a,b\right)$					
Groups	**“1”**	**“2”**	**“1”**	**“3”**	**“2”**	**“3”**
$Se\left(JT\right),\%$	N=24;n=2; **92.3**		N=25 ;n=1; **96.2**		N=25 ;n=1; **96.2**	
$Se\left(MMT\right),\%$	N=21;n=5; **80.8**		N=24;n=2; **92.3**		N=23 ;n=3; **88.5**	
$Sp\left(JT\right),\%$	H=23 ;h=3; **88.5**		H=25;h=1; **96.2**		H=24 ;h=2; **92.3**	
$Sp\left(MMT\right),\%$	H=20 ;h=6; **76.9**		H=22 ;h=4; **84.6**		H=21 ;h=5; **80.8**	
$Ac\left(JT\right),\%$	**90.4**		**96.2**		**94.2**	
$Ac\left(MMT\right),\%$	**78.8**		**88.5**		**84.6**	
Z3;4π8	$LD\left(a,b\right)$					
Groups	**“1”**	**“2”**	**“1”**	**“3”**	**“2”**	**“3”**
$Se\left(JT\right),\%$	N=22;n=4; **84.6**		N=25;n=1; **96.2**		N=23 ;n=3; **88.5**	
$Se\left(MMT\right),\%$	N=18 ;n=8; **69.2**		N=20 ;n=6; **76.9**		N=17;n=9; **65.4**	
$Sp\left(JT\right),\%$	H=21 ;h=5; **80.8**		H=23 ;h=3; **88.5**		H=24 ;h=2; **92.3**	
$Sp\left(MMT\right),\%$	H=19 ;h=7; **73.1**		H=20 ;h=6; **76.9**		H=16 ;h=10; **61.5**	
$Ac\left(JT\right),\%$	**82.7**		**92.3**		**90.4**	
$Ac\left(MMT\right),\%$	**71.2**		**76.9**		**63.5**	
Z3;4π8	$CD\left(a,b\right)$					
Groups	**“1”**	**“2”**	**“1”**	**“3”**	**“2”**	**“3”**
$Se\left(JT\right),\%$	N=23;n=3; **88.5**		N=24;n=2; **92.3**		N=24;n=2; **92.3**	
$Se\left(MMT\right),\%$	N=19;n=7; **73.1**		N=20;n=6; **76.9**		N=19 ;n=7; **73.1**	
$Sp\left(JT\right),\%$	H=23;h=3; **88.5**		H=25;h=1; **96.2**		H=24 ;h=2; **92.3**	
$Sp\left(MMT\right),\%$	H=18 ;h=8; **69.2**		H=19 ;h=7; **73.1**		H=18 ;h=8; **69.2**	
$Ac\left(JT\right),\%$	**88.5**		**94.2**		**92.3**	
$Ac\left(MMT\right),\%$	**71.2**		**75**		**71.2**	

Here JT—Jones matrix theziography; MMT—Mueller matrix tomography.

## Data Availability

The original contributions presented in the study are included in the article, further inquiries can be directed to the corresponding authors.
